# Connecting moral leadership to work engagement in young and middle-aged Chinese university teachers

**DOI:** 10.3389/fpsyg.2026.1810228

**Published:** 2026-03-26

**Authors:** Haibo Liu, Yan Cheng

**Affiliations:** 1 Dezhou Information Engineering Secondary Vocational School, Dezhou, China; 2 School of General Education, Shandong Huayu University of Technology, Dezhou, China

**Keywords:** moral leadership, professional identity, self-determination theory, university teachers, work engagement

## Abstract

This study explores the psychological mechanism linking moral leadership to the work engagement of young university teachers. Drawing on Self-determination Theory, we tested a model where professional identity—comprising efficacy, belonging, and values—mediates this relationship. Using questionnaire data from 285 teachers analyzed with structural equation modeling, we found moral leadership significantly enhanced all three dimensions of professional identity. While professional efficacy and values positively predicted work engagement, professional belonging did not show a direct impact. Most importantly, the results confirmed a complete mediation: the direct influence of moral leadership on work engagement was non-significant, indicating its effect is entirely channeled through professional identity. This research reveals the core path through which moral leadership operates in higher education, confirming that satisfying teachers’ psychological needs via identity enhancement is key to fostering engagement, and providing clear implications for university management.

## 1 Introduction

In the knowledge economy era, university faculty serve as the core force driving knowledge innovation, and their work engagement directly impacts the quality and development of higher education. Consequently, how to effectively stimulate faculty Work Engagement (WE) has become a critical issue of shared concern among academia and practitioners. Work engagement is defined as a positive and fulfilling psychological state related to work, manifested in three dimensions: vigor, dedication, and absorption (Schaufeli et al., 2006; Yu et al., 2025). High levels of work engagement not only enhance individual performance but also stimulate faculty innovation, thereby injecting sustained momentum into organizational development (Zhang B. et al., 2025; Zhang Y. et al., 2025). However, under mounting professional pressures, effectively stimulating and sustaining faculty work engagement faces significant challenges.

Among numerous antecedent variables influencing work engagement, leadership has been proven to be a key factor in shaping the organizational climate and influencing employee attitudes and behaviors (Xia et al., 2024; Duan, 2012). Particularly within China’s educational context, which emphasizes “the dignity of the teaching profession” and “setting an exemplary standard,” a leadership style rooted in local culture—Moral Leadership (ML)—may offer greater explanatory power. Proposed based on the triadic model of paternalistic leadership, ML specifically refers to leaders influencing subordinates through their own noble character, integrity, and exemplary conduct (Brown and Trevino, 2006; Zhu et al., 2025). For the teaching profession, which highly values moral exemplars, moral leadership should theoretically resonate more deeply and foster greater identification (Qu et al., 2025; Guan and Xu, 2020). Extensive empirical research has confirmed the positive impacts of moral leadership on organizations, such as promoting organizational citizenship behavior, enhancing knowledge sharing (Su et al., 2021, 2022), and inhibiting counterproductive behavior (Jin et al., 2022). However, most of these studies have focused on direct effects. The underlying psychological mechanisms—specifically, how moral leadership transforms moral strength into intrinsic work enthusiasm among teachers—remain to be systematically elucidated.

To bridge this gap, this study introduces professional identity as a crucial psychological bridge. Leadership behaviors must be transformed into employees’ intrinsic motivation by altering their self-perception. This study conceptualizes professional identity as a multidimensional construct comprising professional efficacy, professional belonging, and professional values. Professional efficacy reflects Bandura’s and Adams (1977) self-efficacy theory; professional belonging represents the emotional identification with a professional community; and professional values signify the recognition of the intrinsic meaning of the profession. When teachers’ professional identity across these three dimensions is activated, they are more likely to perceive their work as part of their self-actualization, thereby spontaneously investing greater energy.

Self-Determination Theory (SDT) provides a robust integrative framework for understanding this underlying psychological mechanism (Deci and Ryan, 2000; Jiang and Lin, 2022). SDT posits that human behavior is driven by three innate, universal psychological needs: competence, belongingness, and autonomy. When an organizational environment—particularly one shaped by leadership—effectively supports and satisfies these fundamental needs, individuals’ intrinsic motivation is activated, leading to positive outcomes such as work engagement (Qasim and Laghari, 2025; Rivkin et al., 2014). In the context of this study, moral leadership, through its distinctive behavioral patterns, creates a supportive environment that fulfills these three core needs for teachers (Yan et al., 2022). Crucially, the three dimensions of professional identity concretely embody these psychological needs within the higher education context. Enhanced professional efficacy corresponds to the satisfaction of the competence need; strengthened professional belonging reflects the fulfillment of the belongingness need; and the alignment of professional values signifies the satisfaction of higher-order autonomy needs, as true autonomy implies alignment between individual actions and deeply held values.

Despite existing research, significant gaps remain. Current literature lacks a comprehensive chain model integrating moral leadership, multidimensional professional identity, and work engagement within a unified SDT framework. Most studies employ broad, singular mediating variables, failing to meticulously analyze the relative importance of different psychological cognitive pathways. To address these gaps, this study constructs an integrated model to systematically examine how moral leadership influences teachers’ professional perceptions and emotional experiences.

Logically, moral leadership profoundly resonates with teachers’ autonomy needs by upholding and communicating noble educational ideals, thereby rendering the core value of “teaching and nurturing” vivid and concrete. This alignment imbues teachers’ work with profound meaning, solidifying their professional values (Shamir et al., 1993; Xing, 2022). Furthermore, through integrity, fairness, and care for subordinates, moral leadership fosters a harmonious team atmosphere (Han et al., 2024), effectively satisfying the need for belongingness and extending organizational trust into a strong sense of professional belonging. Finally, such leaders fulfill teachers’ competence needs through trust and empowerment. By acting as professional models and supporting growth, leaders bolster educators’ confidence, directly enhancing their professional efficacy.

Subsequently, these activated dimensions of professional identity serve as vital psychological drivers for work engagement. Teachers with high professional efficacy believe in their ability to accomplish tasks, making them more willing to invest effort and embrace challenges. Professional belonging provides a powerful social support resource that buffers work stress and enhances psychological resilience, driving individuals to strive toward shared community goals (Yu and Xie, 2025). Moreover, when professional values are affirmed, work becomes a source of intrinsic fulfillment, constituting the most enduring energy for work engagement (Bakker, 2015; Zhang and Zhao, 2026). In essence, moral leadership does not directly “command” teachers to engage; rather, it subtly shapes their intrinsic professional identity through a “gentle and unobtrusive” approach, making professional identity a core multidimensional mediator (Li et al., 2024). Nevertheless, considering that a leader’s personal charisma may still exert direct motivational effects on subordinates’ work states (Cai et al., 2022; May et al., 2003; Yu et al., 2025), examining the direct effect remains theoretically necessary.

Based on the above theoretical analysis, this study proposes the following hypotheses: Hypothesis 1 (H1a, H1b, H1c) posits that moral leadership positively influences teachers’ professional values, professional belonging, and professional efficacy, respectively. Hypothesis 2 (H2a, H2b, H2c) proposes that professional efficacy, professional belonging, and professional values positively influence work engagement, respectively. Hypothesis 3 (H3) suggests that moral leadership positively influences teachers’ work engagement. Finally, Hypothesis 4 (H4a, H4b, H4c) posits that professional values, professional belonging, and professional efficacy play mediating roles between moral leadership and teachers’ work engagement.

*H1a*: Moral leadership positively influences teachers’ professional values.

*H1b*: Moral leadership positively influences teachers’ professional belonging.

*H1c*: Moral leadership positively influences teachers’ professional self-efficacy.

*H2a*: Teachers’ professional efficacy positively influences their work engagement.

*H2b*: Teachers’ professional belonging positively influences their work engagement.

*H2c*: Teachers’ professional values positively influence their work engagement.

*H3*: Moral leadership positively influences teachers’ work engagement.

*H4a*: Professional values play an mediating role between moral leadership and teachers’ work engagement.

*H4b*: The sense of professional belonging plays an mediating role between moral leadership and teachers’ work engagement.

*H4c*: Professional efficacy plays an mediating role between moral leadership and teachers’ work engagement.

## 2 Materials and methods

### 2.1 Research model

**Figure 1:**
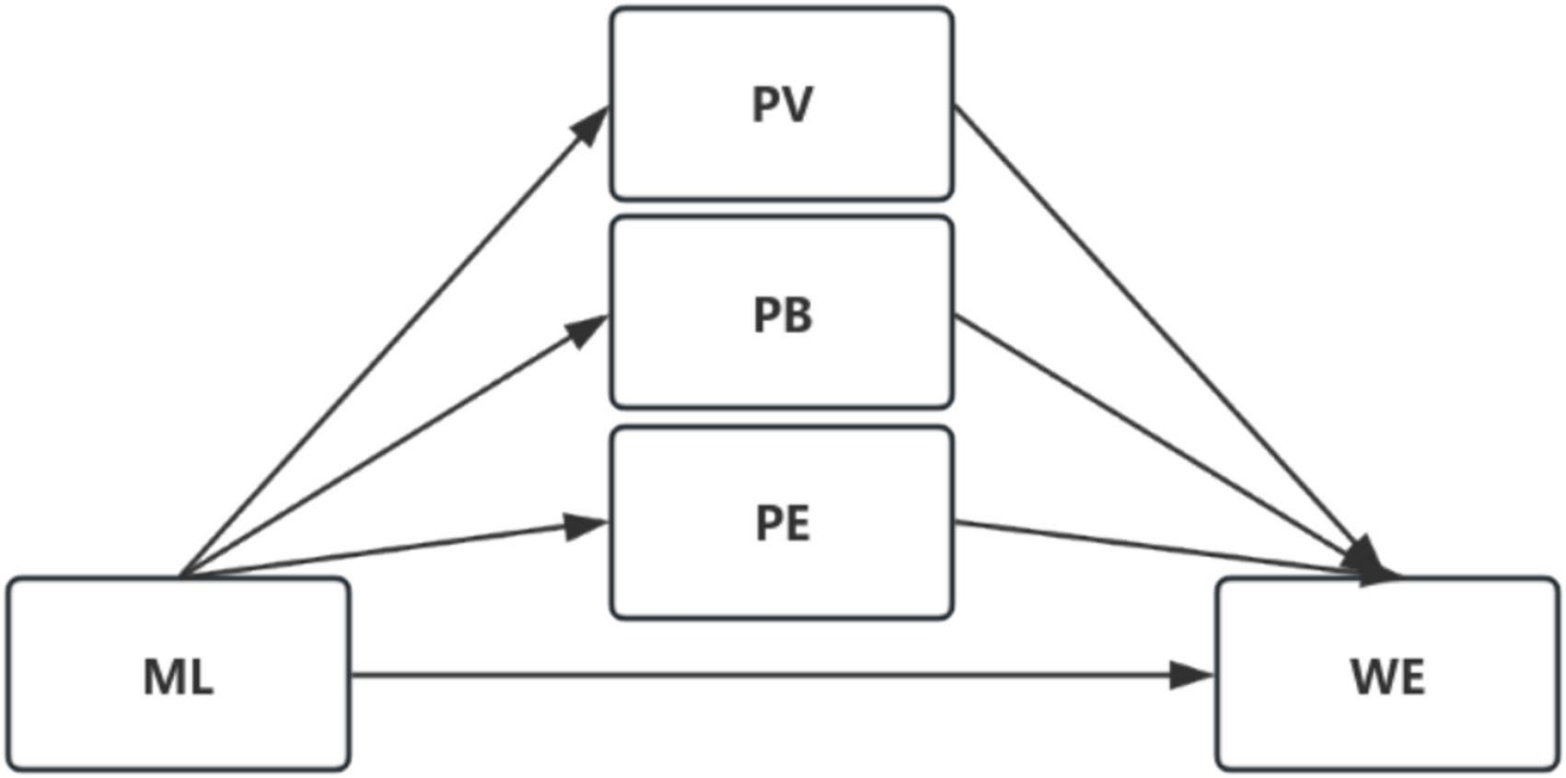
Theoretical model connecting moral leadership to work engagement. ML, moral leadership; PV, professional values; PB, professional belonging; PE, professional efficacy; WE, work engagement. The solid arrows indicate the proposed direct effects, while the pathways through PV, PB, and PE indicate the proposed multidimensional mediating roles. Source: Created by the authors.

Based on the theoretical foundation of Self-Determination Theory and the literature review discussed above, this study constructs a conceptual model to systematically examine the relationships among moral leadership, the multidimensional components of professional identity, and work engagement. The theoretical model is illustrated in Figure 1.

### 2.2 Participants and procedure

This study aims to explore how moral leadership enhances university faculty members’ work engagement by fostering professional identity. A questionnaire survey was conducted among 310 university teachers in China, yielding 285 valid samples after eliminating incomplete or unengaged responses.

Demographic analysis reveals distinct group characteristics relevant to the study’s context: female faculty members form the majority (74.7%), and over 90% of the participants are young or middle-aged teachers aged 50 and below (with 50.1% aged 30 and below, and 46.0% aged between 31 and 50). Furthermore, junior and intermediate-level faculty holding lecturer titles or below constitute the overwhelming majority (82.5%), while associate professors and above account for 17.5%. This highly concentrated sample profile aligns closely with the core research topic, as young and middle-aged faculty in the early or developmental stages of their careers are at a critical juncture for shaping and consolidating their professional identity. They are theoretically more receptive to the exemplary guidance and inspirational appeal of moral leadership, and their work engagement is highly malleable. As shown in Table 1.

**Table 1:** Descriptive statistical analysis of samples.

Type	Variable	Frequency	Percentage (%)
Gender	Male	72	25.3
	Female	213	74.7
Age	30 and below	143	50.1
	31–50	131	46.0
	Over 50	11	3.9
Title	Lecturer and below	235	82.5
	Associate Professor and above	50	17.5

### 2.3 Ethical considerations and informed consent

To ensure strict adherence to ethical research standards, prior to data collection, informed consent was obtained from all participating teachers. Before accessing the survey items, participants were presented with an introductory page that clearly stated the academic purpose of the study. It assured participants that their involvement was entirely voluntary, completely anonymous, and that they had the right to withdraw from the study at any time without any adverse consequences or penalties. They were also informed that all collected data would be securely stored, kept strictly confidential, and used exclusively for academic research purposes. Only those who read this information and explicitly checked the “I consent to participate” box were allowed to proceed to the questionnaire.

### 2.4 Measures

All variables measured in this study were assessed using well-established, standardized scales, with appropriate semantic modifications to suit the localized context of Chinese higher education. All measurements employed a five-point Likert scale, ranging from “1” (Strongly Disagree) to “5” (Strongly Agree).

*Moral leadership*: The measurement of moral leadership was adapted from the scale developed by Li and Shi (2005), which assesses leaders’ exemplary conduct and ethical integrity.

*Professional identity*: The multidimensional measurement of professional identity referenced the work of Li and Yan (2018), covering three distinct sub-dimensions: professional efficacy, professional belonging, and professional values.

*Work engagement*: Work engagement was evaluated based on the established scale by Schaufeli et al. (2006), capturing the vigor, dedication, and absorption of the faculty members in their work environment.

### 2.5 Statistical analysis

This study employed SPSS 27.0 and AMOS 26.0 statistical software for empirical data analysis. The analytical procedure comprised three main steps. First, reliability and validity tests were conducted to evaluate the measurement tools’ quality, including assessments of internal consistency reliability, composite reliability, discriminant validity, and convergent validity. Second, Confirmatory Factor Analysis (CFA) and Structural Equation Modeling (SEM) were utilized to examine the path relationships among variables and their significance levels. Finally, the Bootstrap sampling method (with 5,000 resamples) was employed to rigorously test the mediating effects and verify the statistical significance of the indirect pathways.

## 3 Results

### 3.1 Normal distribution

To ensure the data met the assumption of normal distribution, statistical tests for skewness and kurtosis were first conducted on all measurement indicators. The results showed skewness values ranged from −1.126 to −0.181, none exceeding the critical value of 3; kurtosis values ranged from −0.963 to 1.062, also not surpassing the upper limit of 10 (Cheng et al., 2022). It can thus be concluded that the data broadly satisfy the assumption of normal distribution. After confirming the data distribution met requirements, this study employed covariance-based structural equation modeling (SEM) to validate the proposed theoretical model.

### 3.2 Common method biases

This study employs both confirmatory factor analysis comparison (CFA comparison) and unmeasured latent construct method (ULMC) approaches to systematically examine common method bias. Specifically, in the CFA comparison analysis, researchers constructed a single-factor model incorporating the method factor (all items loading onto a single latent variable) and a five-factor theoretical model for comparative analysis (Guo et al., 2025; Ke et al., 2025). As shown in Table 2, the five-factor model demonstrated significantly superior fit indices compared to the single-factor model. This indicates that the data in this study were minimally affected by common method bias, and the model effectively captured the true relationships among the latent variables.

**Table 2:** Changes of CFA comparison model.

Model	*χ*^2^	df	△*χ*^2^	△df	*p*
one-factor	1175.013	135	902.155	10	0.000
Five-factor	272.858	125			

To conduct a more rigorous assessment of common method bias, this study implemented a test for unmeasured latent method construct (ULMC). This procedure was achieved through model comparison: we contrasted the benchmark confirmatory factor analysis model (M1) with an alternative two-factor model (M2). Model M2 adds a latent factor representing the common method effect to M1, with all observed indicators loading onto both their theoretical construct factors and this common method factor. According to established criteria (Wang and Li, 2019), if the model fit does not substantially improve after introducing the method factor (i.e., △GFI/CFI/TLI < 0.1, △RMSEA/SRMR < 0.05), the impact of common method bias can be considered negligible. As shown in Table 3, the model comparison results in this study fully meet this criterion, providing further statistical evidence for the robustness of the research conclusions.

**Table 3:** ULMC test results.

Model	*χ*^2^	df	*χ*^2^/df	GFI	CFI	TLI	RMSEA	SRMR	△GFI	△CFI	△TLI	△RMSEA	△SRMR
M1	272.858	125	2.183	0.902	0.961	0.952	0.065	0.0396	0.010	0.005	0.004	−0.003	0.005
M2	243.623	117	2.082	0.912	0.966	0.956	0.062	0.045					

### 3.3 Confirmatory factor analysis

Based on the reliability and convergent validity test results in Table 4, all dimensions demonstrated satisfactory measurement performance. First, Cronbach’s *α* coefficients for all dimensions exceeded 0.7, indicating strong internal consistency (Ke et al., 2025). Second, composite reliability (CR) and average variance extracted (AVE) also yielded favorable metrics, with most dimensions exhibiting CR values above 0.8 and AVE exceeding 0.6. This validates the model’s robust convergent validity, confirming its ability to effectively reflect the intrinsic structure of each latent variable (Zhang and Zhao, 2026). Furthermore, all standardized path coefficients for the items were statistically significant (*Z* values > 7, *p* values < 0.001), further demonstrating the reliability and validity of the measurement model.

**Table 4:** Reliability and convergence validity test.

Constructs	Indicators	Unstandardized coefficients	SE	*Z*-value	*p* value	Item loadings	Croubach’s α	CR	AVE
ML	ML1	1				0.85	0.899	0.901	0.695
	ML2	1.007	0.062	16.366	***	0.811			
	ML3	0.975	0.062	15.717	***	0.789			
	ML4	1.06	0.057	18.492	***	0.881			
PV	PV1	1				0.786	0.887	0.889	0.668
	PV2	1.017	0.067	15.226	***	0.827			
	PV3	1.152	0.07	16.472	***	0.881			
	PV4	1.024	0.073	13.94	***	0.77			
PB	PB1	1				0.876	0.898	0.899	0.749
	PB2	1.069	0.054	19.906	***	0.883			
	PB3	0.976	0.054	18.166	***	0.836			
PE	PE1	1				0.709	0.845	0.858	0.671
	PE2	1.009	0.075	13.485	***	0.847			
	PE3	1.14	0.081	14.075	***	0.89			
WE	WE1	1				0.721	0.870	0.875	0.638
	WE2	1.01	0.076	13.263	***	0.825			
	WE3	1.006	0.074	13.622	***	0.849			
	WE4	0.969	0.076	12.771	***	0.793			

According to the discriminant validity test results in Table 5, all dimensions demonstrate strong discriminative power. Specifically, the correlation coefficients for dimensions such as work engagement, career values, career commitment, career efficacy, and moral leadership are all lower than the square root of the AVE for their respective dimensions. This indicates that latent variables can be clearly distinguished from one another, avoiding excessive overlap between latent variables (Zhang B. et al., 2025; Zhang Y. et al., 2025). Therefore, the model demonstrates excellent discriminant validity and can effectively differentiate between different research constructs.

**Table 5:** Discriminatory validity test.

Dimension	WE	PE	PB	PV	ML
WE	**0.798**				
PE	0.726	**0.819**			
PB	0.654	0.772	**0.865**		
PV	0.683	0.801	0.808	**0.817**	
ML	0.417	0.502	0.619	0.617	**0.834**

The diagonal bold part is the square root of the AVE.

### 3.4 Hypothesis test

The model fit indices met ideal standards across multiple measures, demonstrating excellent model fit. Specifically, the *χ*^2^/df ratio was 2.183 (<3.00), the RMSEA value was 0.065 (<0.08), and the CFI, IFI, and TLI indices were 0.961, 0.961, and 0.952 (all >0.90), respectively. SRMR was 0.045 (<0.08), while GFI and AGFI were 0.902 and 0.865, respectively (both >0.80). These data fully indicate that the model possesses high fit quality and can effectively explain the observed data (Wang et al., 2022).

Regarding hypothesis testing for the structural model, as shown in Table 6, the direct effect paths for research hypotheses H1a, H1b, H1c, H2a, and H2c all exhibited significant positive effects and were supported. However, the regression coefficients for the paths H2b and H3 did not reach statistical significance and were not validated.

**Table 6:** Hypothesis test results of the model.

Hypothesized relationship	Non-standardized coefficients	standard error	*Z* price	*p* price	Standardization coefficient
H1a: ML → PV	0.581	0.056	10.403	***	0.704
H1b: ML → PB	0.637	0.059	10.721	***	0.701
H1c: ML → PE	0.551	0.058	9.441	***	0.604
H2a: PE → WE	0.697	0.074	9.433	***	0.774
H2b: PB → WE	0.101	0.064	1.584	0.113	0.112
H2c: PV → WE	0.161	0.072	2.243	0.025	0.162
H3: ML → WE	−0.108	0.085	−1.268	0.205	−0.132

### 3.5 Mediation effect test

To delve into the pathways through which moral leadership influences work engagement, this study focuses on the core mediating role of professional identity. Using the Bootstrap method, we specifically examined the indirect roles played by the three core dimensions of professional identity—professional efficacy, professional belonging, and professional values. Following the recommendations of Preacher and Hayes (Jia et al., 2024; Cai, 2021), we set 1,000 resamples to estimate 95% confidence intervals. We deemed the mediating effect significant only when the confidence interval completely excluded zero.

This study first examined the mediating role of professional values between moral leadership and work engagement. The results indicate that professional values serve as a crucial bridge connecting moral leadership to teacher work engagement. As shown in Table 7, the effect size of the indirect path (moral leadership → professional values → work engagement) was 0.345, with a 95% confidence interval [0.244, 0.495] entirely excluding zero, indicating this path was significantly established. Simultaneously, after controlling for professional values, the direct effect of moral leadership on work engagement (−0.005) became non-significant, with its 95% confidence interval [−0.122, 0.108] encompassing zero. This pattern of results clearly reveals the full mediating role of professional values: moral leadership does not directly enhance teachers’ work engagement but instead achieves its positive impact entirely through strengthening teachers’ professional values—a core psychological resource.

**Table 7:** Mediating effects of professional values.

Path relationship	Point estimate	Product of coefficient		Bootstrapping 1,000 times 95% CI			
				Bias-corrected		Percentile	
		SE	*Z*-value	Lower	Upper	Lower	Upper
Indirect effect							
ML → PV → WE(IE)	0.345	0.063	5.476	0.244	0.495	0.233	0.482
Direct effect							
ML → WE(DE)	−0.005	0.06	−0.083	−0.122	0.108	−0.117	0.112
Total effect							
(IE + DE)	0.339	0.066	5.136	0.21	0.469	0.21	0.473

Second, continuing the exploration of underlying psychological mechanisms, this study further examined the role of professional belonging in mediating the relationship between moral leadership and teacher work engagement. As shown in Table 8, Bootstrap analysis revealed another crucial influence pathway: the indirect effect of moral leadership fostering teachers’ professional belonging on work engagement was significant, with an effect size of 0.316 and a 95% confidence interval [0.211, 0.480] that completely excluded zero. Simultaneously, when professional belonging was included in the analysis, the direct effect of leadership behavior on work engagement (0.024) also became insignificant, with its confidence interval [−0.103, 0.146] clearly crossing zero. This pattern of results again points to a full mediation effect. It indicates that the positive influence of moral leadership is not achieved through direct commands or demands, but rather by fostering an organizational atmosphere where teachers feel accepted and recognized. This, in turn, strengthens their sense of belonging and identification with the teaching profession. It is precisely this deep-seated sense of belonging that ultimately drives their wholehearted commitment to their work.

**Table 8:** Mediating effects of profession belonging.

Path relationship	Point estimate	Product of coefficient		Bootstrapping 1,000 times 95% CI			
				Bias-corrected		Percentile	
		SE	*Z*-value	Lower	Upper	Lower	Upper
Indirect effect							
ML → PB → WE(IE)	0.316	0.065	4.862	0.211	0.48	0.205	0.463
Direct effect							
ML → WE(DE)	0.024	0.061	0.393	−0.103	0.146	−0.107	0.141
Total effect							
(IE + DE)	0.341	0.066	5.167	0.21	0.47	0.212	0.473

To delve deeper into the cognitive and affective mechanisms influencing teachers’ work engagement, this study examined the mediating role of professional efficacy and revealed a robust cognitive pathway. As shown in Table 9, the indirect effect of moral leadership on work engagement through professional efficacy is not only positive but highly significant, with an effect size of 0.348 and a 95% confidence interval [0.246, 0.477] that completely excludes zero. Crucially, once this efficacy-building pathway was incorporated into the model, the direct link between leadership behavior and teacher work engagement disappeared. Its effect size dropped to a negligible −0.003, with a confidence interval [−0.117, 0.093] that included zero. This typical result pattern clearly demonstrates the full mediating effect of professional efficacy. This strongly indicates that leaders’ moral exemplification does more than merely foster a positive environment; it resonates with the core meaning of the teaching profession. By reinforcing and aligning with teachers’ professional efficacy, leadership behaviors imbue work with a deeper sense of purpose and mission—the primary driving force behind their wholehearted commitment.

**Table 9:** Mediating effects of profession efficacy.

Path relationship	Point estimate	Product of coefficient		Bootstrapping 1,000 times 95% CI			
				Bias-corrected		Percentile	
		SE	*Z*-value	Lower	Upper	Lower	Upper
Indirect effect							
ML → PE → WE(IE)	0.348	0.059	5.898	0.246	0.477	0.244	0.469
Direct effect							
ML → WE(DE)	−0.003	0.052	−0.058	−0.117	0.093	−0.114	0.098
Total effect							
(IE + DE)	0.345	0.065	5.308	0.215	0.474	0.219	0.475

## 4 Discussion

### 4.1 Contributions to existing literature

This study makes several critical contributions to the existing literature on educational leadership and organizational psychology, particularly within the high-pressure context of contemporary higher education. First, while previous studies have largely confirmed the positive organizational outcomes of moral leadership (Jiatong et al., 2022), they have predominantly treated professional identity as a monolithic and static construct. By decomposing professional identity into a multidimensional framework—comprising professional efficacy, professional belonging, and professional values—this study provides a much more nuanced understanding of the cognitive and affective pathways through which ethical leadership operates.

Second, this research significantly advances the contextual application of Self-Determination Theory (SDT). Academic work is characterized by high intellectual autonomy and significant psychological stress, especially for young and middle-aged faculty facing rigorous tenure-track evaluations. By aligning the three dimensions of professional identity with SDT’s core psychological needs (competence, relatedness, and autonomy), this study theoretically bridges external leadership behaviors with the internal intrinsic motivational structures of academics. Finally, demonstrating the full mediation effect of multidimensional professional identity adds critical depth to our understanding of faculty engagement, proving empirically that external moral leadership cannot dictate engagement directly; rather, it must be internalized into the faculty’s professional self-perception to generate sustained work engagement.

### 4.2 Theoretical implications

The empirical results offer profound theoretical insights, particularly regarding the differential impacts of the professional identity dimensions. The findings confirm that moral leadership does not rely on formal authority or coercive metrics to compel engagement. Instead, by modeling academic integrity, demonstrating fairness in resource allocation, and exhibiting genuine care, ethical leaders cultivate an environment that fulfills teachers’ intrinsic psychological needs.

A particularly salient finding is the differential impact of the identity dimensions on work engagement. Professional efficacy (H2a) and professional values (H2c) emerged as strong direct predictors of work engagement. This aligns with the nature of academic labor: scholarly engagement requires immense cognitive vigor and absorption (Schaufeli et al., 2006; Xing, 2022), which are directly fueled by a strong belief in one’s academic capabilities (efficacy) and a deep resonance with the ideological meaning of education (values).

Interestingly, while professional belonging significantly mediated the relationship between moral leadership and work engagement in the bootstrap analysis (H4b), its direct predictive effect on work engagement in the structural model did not reach statistical significance (H2b unsupported). This nuanced, seemingly contradictory finding actually prompts valuable theoretical reflection. Work engagement specifically captures task-focused vitality and dedication. While a strong sense of belonging within a department fosters emotional safety, prevents burnout, and reduces turnover intention (Mesdaghinia et al., 2023), it may not immediately or directly translate into the high-intensity task engagement required for publishing papers or delivering demanding lectures. Furthermore, this phenomenon can be largely attributed to the highly individualistic and autonomous nature of academic labor. Because researchers frequently work independently on highly specialized topics, institutional belonging can sometimes become decoupled from personal, solitary task engagement. Belonging acts as a crucial foundational bridge (as shown in the mediation test), creating a safe baseline from which efficacy and values can thrive, but it is “meaning-driven” (values) and “capability-driven” (efficacy) motivations that serve as the direct psychological catalysts for intense work engagement.

Furthermore, the rejection of the direct effect of moral leadership on work engagement after introducing the mediators (H3 unsupported) strongly corroborates the fundamental premise of SDT. It highlights a vital theoretical boundary: moral leadership is a distal environmental antecedent, not a proximal psychological trigger. Without the internalization process of building a robust professional identity, even the most ethical leadership will fail to elicit deep work engagement from faculty.

### 4.3 Practical implications

The findings yield actionable, specific insights for university administrators and human resource management in higher education. First, amidst the prevailing “performance-driven” and metric-heavy academic culture, universities must consciously integrate “value-shaping” leadership into their management structures. Department heads and deans should prioritize ethical character, ensure absolute transparency and fairness in the distribution of research grants and lab spaces, and fiercely protect the academic autonomy of young scholars. Such moral leadership creates a psychologically safe environment that acts as the primary catalyst for intrinsic motivation.

Second, institutions should strategically design policies that specifically nurture the multidimensional professional identity of their faculty:

*Empowerment to boost efficacy*: Universities should move beyond mere evaluation and provide young faculty with substantial developmental support. Implementing targeted mentorship programs, providing seed funding for early-career research, and offering pedagogical training can rapidly build the professional confidence and competence of junior faculty.

*Care to strengthen belonging*: Although its direct impact on immediate task engagement is nuanced, belonging is vital for long-term retention and mental well-being. Departments should dismantle academic silos and foster a collaborative “scholarly community” culture through regular, informal academic salons and peer-support networks.

*Value-shaping to inspire dedication*: Institutions must consistently reinforce the noble mission of higher education. By reforming evaluation metrics to reward not only high-impact publications but also pedagogical excellence and ethical academic conduct, universities can help faculty rediscover and deepen their resonance with the core value of “cultivating virtue through education.”

### 4.4 Limitations and future prospects

Despite its robust theoretical and practical contributions, this study has several limitations that warrant future research. First, the cross-sectional design constrains the ability to establish definitive, long-term causal relationships. For instance, reciprocal causality—where highly engaged and successful teachers might naturally perceive their academic leaders more positively—cannot be entirely ruled out. Future studies should employ longitudinal or cross-lagged panel designs to capture the dynamic interplay among these variables over an extended academic cycle.

Second, the reliance on self-reported survey data introduces the potential for social desirability bias and common method bias (Podsakoff et al., 2003; Zhang and Zhao, 2026). Particularly in hierarchical academic environments, evaluating “moral leadership” and “work engagement” involves sensitive self-presentation. Participants might unconsciously or deliberately inflate their scores to conform to social norms, meet institutional expectations, or appear more highly committed. This tendency could artificially inflate the correlations between variables and mask the true psychological dynamics. While rigorous statistical tests indicated it was not a severe issue in this dataset, future research could significantly benefit from multi-source data, such as incorporating peer evaluations, student feedback, or objective academic performance metrics.

Third, the demographic distribution of our sample—predominantly female (74.7%) and junior-level faculty (82.5%)—limits the broader generalizability of the findings. While this demographic is highly relevant for examining the formative stages of professional identity, the results may not fully represent the psychological dynamics of senior academic teams or male-dominated disciplines. Future research should explore potential moderating variables across a more balanced demographic sample and different cultural contexts (e.g., Western academic institutions) to further enrich and validate this integrated theoretical model.

## 5 Conclusion

In conclusion, based on the integrative framework of Self-Determination Theory, this study systematically unravels the complex psychological mechanisms connecting moral leadership to work engagement among young and middle-aged university teachers. In direct review of our hypotheses, the empirical results strongly support that moral leadership significantly and positively enhances teachers’ professional values (H1a), professional belonging (H1b), and professional efficacy (H1c). Furthermore, professional efficacy (H2a) and professional values (H2c) significantly predict work engagement, whereas the direct effect of professional belonging on work engagement (H2b) was not supported. Crucially, after introducing the multidimensional mediators, the direct effect of moral leadership on work engagement became entirely insignificant (H3 is not supported). Bootstrap analyses confirmed that professional values (H4a), professional belonging (H4b), and professional efficacy (H4c) all serve as vital full mediators. Ultimately, this study concludes that moral leadership operates entirely through a full mediation mechanism; it elevates faculty engagement fundamentally by satisfying intrinsic psychological needs and solidifying a multidimensional professional identity. These findings offer robust theoretical evidence and highly actionable guidelines for motivating and sustaining academic talent in contemporary higher education environments.

## Data Availability

The original contributions presented in the study are included in the article/supplementary material, further inquiries can be directed to the corresponding author.

## References

[ref1] Bakker A. B. (2015). Daily fluctuations in work engagement. Eur. Psychol. 19:227. doi: 10.1027/1016-9040/A000160

[ref2] Bandura A. Adams N. E. (1977). Analysis of self-efficacy theory of behavioral change. Cogn. Ther. Res. 1, 287–310. doi: 10.1007/bf01663995

[ref3] Brown M. E. Trevino L. K. (2006). Ethical leadership: a review and future directions. Leadersh. Q. 17, 595–616. doi: 10.1016/j.leaqua.2006.10.004

[ref4] Cai H. (2021). Exploring the relationship between teachers’ online teaching readiness and students’ learning outcomes: the mediating roles of learner control and academic emotions. J. East China Norm. Univ. (Educ. Sci.) 39, 27–37. doi: 10.16382/j.cnki.1000-5560.2021.07.003 (in Chinese)

[ref5] Cai Z. Zhu J. Tian S. (2022). Preservice teachers’ teaching internship affects professional identity: self-efficacy and learning engagement as mediators. Front. Psychol. 13:1070763. doi: 10.3389/fpsyg.2022.1070763, 36532965 PMC9748549

[ref6] Cheng J. Sun X. Lu J. He Y. (2022). How ethical leadership prompts employees’ voice behavior? The roles of employees’ affective commitment and moral disengagement. Front. Psychol. 12:732463. doi: 10.3389/fpsyg.2021.732463, 35126225 PMC8810509

[ref7] Deci E. L. Ryan R. M. (2000). The ‘what’ and ‘why’ of goal pursuits: human needs and the self-determination of behavior. Psychol. Inq. 11, 227–268. doi: 10.1207/S15327965PLI1104_01

[ref8] Duan J. (2012). The impact of paternalistic leadership on employee voice behavior: the mediating mechanism of psychological safety. Manag. Rev. 24, 109–116. doi: 10.14120/j.cnki.cn11-5057/f.2012.10.002 (in Chinese)

[ref9] Guan S. Xu S. (2020). An empirical study on the leadership behavior structure of university discipline leaders. Jiangsu High. Educ. 11, 55–60. doi: 10.13236/j.cnki.jshe.2020.11.009

[ref10] Guo T. Zhang D. Yang J. Xia J. (2025). Exploring how the ambidextrous leadership influences knowledge workers innovative behavior: a two stage SEM-ANN analysis. Front. Psychol. 16:1560726. doi: 10.3389/fpsyg.2025.1560726, 40547580 PMC12179793

[ref11] Han Z. Xi Y. Qin J. Ren Z. Hu F. (2024). Employee honesty-humility and workplace deviance behavior: from the perspective of trait activation theory. Psychol. Sci. 47, 178–186. doi: 10.16719/j.cnki.1671-6981.20240121 (in Chinese)

[ref12] Jia H. Wu J. Lu Y. (2024). Research on the mechanism of environmental responsibility behavior of residents in agricultural cultural heritage tourism destinations. Economic Problems. 9, 111–120. doi: 10.16011/j.cnki.jjwt.2024.09.002 (in Chinese)

[ref13] Jiang R. Lin X. (2022). Trickle-down effect of moral leadership on unethical employee behavior: a cross-level moderated mediation model. Pers. Rev. 51, 1362–1385. doi: 10.1108/PR-04-2020-0257

[ref14] Jiatong W. Wang Z. Alam M. Murad M. Gul F. Gill S. A. (2022). The impact of transformational leadership on affective organizational commitment and job performance: the mediating role of employee engagement. Front. Psychol. 13:831060. doi: 10.3389/fpsyg.2022.831060, 35465551 PMC9019157

[ref15] Jin S. Zhu X. Fu X. Wang J. (2022). Family supportive leadership and counterproductive work behavior: the roles of work-family conflict, moral disengagement and personal life attribution. Front. Psychol. 13:906877. doi: 10.3389/fpsyg.2022.906877, 35693528 PMC9184795

[ref16] Ke Y. Liu L. Gu M. (2025). Paternalistic leadership and counterproductive work behavior: mediating role of leader identification and moderating effect of traditionality in Chinese generation Z employees. Front. Psychol. 16:1587525. doi: 10.3389/fpsyg.2025.1587525, 40697732 PMC12279840

[ref17] Li R. Chen Q. Li H. Li Y. (2024). Correlation between professional identity and work engagement among nursing interns. Mil. Nurs. 41, 64–67. (in Chinese)

[ref18] Li C. Shi K. (2005). The structure and measurement of transformational leadership. Acta Psychol. Sin. 37, 97–105. (in Chinese)

[ref19] Li X. Yan H. (2018). Model construction and scale development of teachers’ professional identity. Teach. Educ. Res. 30, 72–81. doi: 10.13445/j.cnki.t.e.r.2018.02.011 (in Chinese)

[ref20] May D. Chan A. Hodges T. Avolio B. (2003). Developing the moral component of authentic leadership. Organ. Dyn. 32, 247–260. doi: 10.1016/S0090-2616(03)00032-9

[ref21] Mesdaghinia S. Eisenberger R. Wen X. Liu Z. Lewis B. A. Qiu F. . (2023). How leaders drive followers’ unethical behavior. J. Manage. 49:01492063221104031. doi: 10.1177/01492063221104031

[ref22] Podsakoff P. M. MacKenzie S. B. Lee J.-Y. Podsakoff N. P. (2003). Common method biases in behavioral research: a critical review of the literature and recommended remedies. J. Appl. Psychol. 88, 879–903. doi: 10.1037/0021-9010.88.5.879, 14516251

[ref23] Qasim M. Laghari A. A. (2025). Belonging through values: ethical leadership, creativity, and psychological safety with ethical climate as a moderator. Front. Psychol. 16:1559427. doi: 10.3389/fpsyg.2025.1559427, 40386677 PMC12082719

[ref24] Qu Y. Liu S. Shao Y. Li G. (2025). Moral leadership and professional learning communities: the mediating role of teachers’ trust in principals and the moderating role of principals’ controlling behavior. Psychol. Sch. 62, 1369–1382. doi: 10.1002/pits.23398

[ref25] Rivkin W. Diestel S. Schmidt K.-H. (2014). The positive relationship between servant leadership and employees’ psychological health: a multi-method approach. Z. Personforsch. 28, 52–72. doi: 10.1688/ZfP-2014-01-Rivkin

[ref26] Schaufeli W. B. Bakker A. B. Salanova M. (2006). The measurement of work engagement with a short questionnaire: a cross-national study. Educ. Psychol. Meas. 66, 701–716. doi: 10.1177/0013164405282471

[ref27] Shamir B. House R. J. Arthur M. B. (1993). The motivational effects of charismatic leadership: a self-concept based theory. Organ. Sci. 4, 577–594. doi: 10.1287/orsc.4.4.577

[ref28] Su X. Jiang X. Xie G. Huang M. Xu A. (2022). How does self-sacrificial leadership foster knowledge sharing behavior in employees? Moral ownership, felt obligation and supervisor-subordinate guanxi. Front. Psychol. 13:910707. doi: 10.3389/fpsyg.2022.910707, 35899007 PMC9309226

[ref29] Su X. Lin W. Wu J. Zheng Q. Chen X. Jiang X. (2021). Ethical leadership and knowledge sharing: the effects of positive reciprocity and moral efficacy. SAGE Open 11:21582440211021823. doi: 10.1177/21582440211021823

[ref30] Wang Y. Li H. (2019). Moral leadership and unethical pro-organizational behavior: a moderated mediation model. Front. Psychol. 10:2640. doi: 10.3389/fpsyg.2019.02640, 31849761 PMC6892784

[ref31] Wang Y. Liu S. Ma J. Yang Q. Wang K. (2022). The effect of negative emotions on eating disorder symptoms in sports majors: the mediating effects of compulsive exercise and self-esteem. Chin. J. Clin. Psychol. 30, 1308–1312. doi: 10.16128/j.cnki.1005-3611.2022.06.009 (in Chinese)

[ref32] Xia W. Fan Y. Bai J. Zhang Q. Wen Y. (2024). The relationship between organizational climate and job satisfaction of kindergarten teachers: a chain mediation model of occupational stress and emotional labor. Front. Psychol. 15:1373892. doi: 10.3389/fpsyg.2024.1373892, 38863665 PMC11165699

[ref33] Xing Z. (2022). English as a foreign language teachers’ work engagement, burnout, and their professional identity. Front. Psychol. 13:916079. doi: 10.3389/fpsyg.2022.916079, 35756227 PMC9218421

[ref34] Yan M. Li S. Wang H. Ma Y. (2022). A study on the mechanism of moral leadership from the perspective of basic need satisfaction. Manag. Rev. 34, 173–184. doi: 10.14120/j.cnki.cn11-5057/f.2022.04.002 (in Chinese)

[ref35] Yu J. Xie C. (2025). Study on the influencing factors and process mechanism of tourist resilience. Tour. Trib.

[ref36] Yu X. Xiong Z. Li W. Zhang H. Li X. Xiao W. (2025). Teacher professional identity on work engagement: the moderating roles of ego-resilience and perceived organizational support. Front. Psychol. 16:1657911. doi: 10.3389/fpsyg.2025.1657911, 41394049 PMC12695751

[ref37] Zhang B. Xu S. Yang C. (2025). The influence of craftsmanship spirit on employees’ innovative behavior: the role of psychological availability and different types of ethical leadership. Technol. Econ. 44, 96–108. (in Chinese)

[ref38] Zhang Y. Zhang J. Hao K. (2025). Boosting work engagement through leader tolerance: the chain mediation role of perceived organizational support and organizational identification. Front. Psychol. 16:1489147. doi: 10.3389/fpsyg.2025.1489147, 40166391 PMC11956503

[ref39] Zhang Y. Zhao D. (2026). How paternalistic leadership influences teachers’ resistance to STEM reform: the mediating role of teachers’ STEM literacy and the moderating role of work engagement. Front. Psychol. 17:1726148. doi: 10.3389/fpsyg.2026.1726148, 41809742 PMC12968017

[ref40] Zhu J. Zhi W. Fang Y. (2025). Ethical leadership, organizational learning, and corporate ESG performance: a moderated mediation model. Int. Rev. Econ. Finance 98:103966. doi: 10.1016/j.iref.2025.103966

